# 

**Published:** 1907-11

**Authors:** 


### BOOKS RECEIVED.

The Diagnosis and Treatment of Diseases of Women, by Harry Sturgeon Crossen, M.D., Clinical Professor of Gynecology in Washington University; Gynecologist to Washington University and Chief of the Gynecological Clinic. Octavo, pp. 813. With 700 illustrations. Saint Louis: C. V. Mosby Medical Book and Publishing Company. 1907.

---

Proceedings of the American Medico-Psychological Association at the sixty-second annual meeting held at Boston, June 12-15, 1906. Charles W. Pilgrim, M.D., Secretary.

---

A Textbook of the Practice of Medicine, for Students and Practitioners, by James Magoffin French, A.M., M.D. Third revised edition: Octavo, 1276 pages, illustrated by numerous engravings in the text and by twenty-five full-page plates by chromo-lithography, photogravure, chromotype, etc. New York: Wm. Wood & Co., (Muslin, $5.50; leather $6.50 net prices).

---

The Pancreas. Its Surgery and Pathology. By A. W. Mayo Robson, D. Sc. (Leeds) F.R.C.S. (Eng.), London, and P. J. Cammidge, M.B. (Lond.) D.P.H. (Camb.) Octavo, pp. 546. Illustrated. Philadelphia and London: W. B. Saunders Co. (Price, $5.00.)

